# Abnormal resting-state cortical coupling in chronic tinnitus

**DOI:** 10.1186/1471-2202-10-11

**Published:** 2009-02-19

**Authors:** Winfried Schlee, Thomas Hartmann, Berthold Langguth, Nathan Weisz

**Affiliations:** 1 University of Konstanz, Konstanz, Department of Psychology, Germany; 2 University of Regensburg, Regensburg, Department of Psychiatry, Bezirksklinikum Regensburg, Germany

## Abstract

**Background:**

Subjective tinnitus is characterized by an auditory phantom perception in the absence of any physical sound source. Consequently, in a quiet environment, tinnitus patients differ from control participants because they constantly perceive a sound whereas controls do not. We hypothesized that this difference is expressed by differential activation of distributed cortical networks.

**Results:**

The analysis was based on a sample of 41 participants: 21 patients with chronic tinnitus and 20 healthy control participants. To investigate the architecture of these networks, we used phase locking analysis in the 1–90 Hz frequency range of a minute of resting-state MEG recording. We found: 1) For tinnitus patients: A significant decrease of inter-areal coupling in the alpha (9–12 Hz) band and an increase of inter-areal coupling in the 48–54 Hz gamma frequency range relative to the control group. 2) For both groups: an inverse relationship (r = -.71) of the alpha and gamma network coupling. 3) A discrimination of 83% between the patient and the control group based on the alpha and gamma networks. 4) An effect of manifestation on the distribution of the gamma network: In patients with a tinnitus history of less than 4 years, the left temporal cortex was predominant in the gamma network whereas in patients with tinnitus duration of more than 4 years, the gamma network was more widely distributed including more frontal and parietal regions.

**Conclusion:**

In the here presented data set we found strong support for an alteration of long-range coupling in tinnitus. Long-range coupling in the alpha frequency band was decreased for tinnitus patients while long-range gamma coupling was increased. These changes discriminate well between tinnitus and control participants. We propose a tinnitus model that integrates this finding in the current knowledge about tinnitus. Furthermore we discuss the impact of this finding to tinnitus therapies using Transcranial Magnetic Stimulation (TMS).

## Background

Patients that suffer from chronic tinnitus complain of an ongoing perception of a phantom sound in the absence of any physical source for it. About 5–15% of the population in western societies experience a phantom tinnitus sound and 1–3% of the population suffer from severe tinnitus that affects their daily life and is accompanied in 50% of the cases by depression, in 40% of the cases by insomnia and about 20% of the patients complain of an important decrease in their quality of life [1,2]. Unfortunately, the underlying mechanisms responsible for the tinnitus perception is currently not known. Tinnitus therapies typically concentrate on coping with the tinnitus but there is no therapy that reliably reduces the perception of tinnitus.

Tinnitus is often accompanied by damage to the peripheral hearing system and a series of plastic changes in the central auditory system are observed in parallel to that. It is thought that a deafferentation of the hearing system triggers a series of reorganization processes at all levels of the auditory system [3]. Indeed, abnormal neuronal activity in tinnitus has been demonstrated for the auditory nerve fibers, the dorsal cochlear nucleus, the inferior colliculus, the primary and the secondary auditory cortex (see a review of this in [3]). Furthermore, it has been found that a dissection of the auditory nerve in tinnitus patients does not lead to relief in tinnitus and most of the patients still experience tinnitus after surgery [4,5]. Thus, there is an agreement that the tinnitus phantom sound is generated in the central nervous system – most likely as a result of the reorganization that is going on in the auditory system after hearing loss.

However, there are also studies that demonstrated tinnitus-related cortical abnormalities outside the auditory system. Using methods as different as Positron Emission Tomography (PET), Voxel Based Morphometry (VBM) and Magnetoencephalography (MEG) differences in cortical activity have been shown for the frontal cortex, the parietal lobe, mesial posterior regions and the subcollosal region including the nucleus accumbens [6-8]. As hypothesized earlier by Jastreboff [9] it might be that tinnitus is generated within the auditory system while non-auditory regions are involved in encoding the conscious percept well as the emotional evaluation of it. This idea also fits with a recently established model of the global neuronal workspace by Deheane and colleagues [10]. This group suggests the existence of workspace neurons that are located mainly in the parietal lobe, the frontal, the cingulate cortex and the sensory systems. In order to form a conscious percept of a stimulus, two conditions are required: First, neuronal activity of the sensory cortex of the respective modality. Second, an entry into the global neuronal workspace and thus long-range coupling between the widely distributed workspace neurons. According to this model, coupling within this fronto-parietal-cingulate network is needed for conscious perception (i.e. awareness of the stimulus). Activity of the sensory areas without this coupling would remain preconscious.

Different brain regions need to communicate with each other in order to integrate information, perform their specific function and distribute information to other brain areas. It has been suggested that this communication is performed by neuronal synchronization between those brain areas and the functional importance of this inter-areal coupling has been shown in several studies [11-18]. In this literature, the terms 'coherence', 'synchrony', and 'coupling' are used with slightly different connotations. To avoid misunderstandings we want to use the term 'coupling' throughout this manuscript to describe the functional interaction between distant Neuronal Cell Assemblies.

The importance of long-range functional coupling has been shown recently by many authors in different fields of neuroscience. For instance, Supp et al. [17] demonstrated different patterns of long-range coupling in the gamma band between visually presented familiar and unfamiliar objects and Miltner et al. [15] found enhanced gamma band coupling during associative learning. Melloni et al.[14] used different masks to manipulate whether a test stimuli was visible or invisible to the participants. They found significant differences of gamma phase locking between the 'visible' and the 'invisible' condition. Hummel and Gerloff [13] showed an increase of alpha band coupling between occipital and left central areas correlates with behavioral performance in a visuotactile integration task. Uhlhaas and colleagues reviewed abnormal neuronal coupling in a large variety of brain disorders, namely schizophrenia, epilepsy, autism, Alzheimer's disease and Parkinson's disease [18]. In a behavioral experiment, Gross et al. [12] showed that changes in the inter-regional coupling vary with changes of the behavioral task demands.

To the best of our knowledge there is currently no study on long-range functional coupling in chronic tinnitus. In previous studies we investigated abnormal power changes in the spontaneous activity of tinnitus patients and found an increase in delta-power (<4 Hz) and a decrease in alpha-power (8–12 Hz). These changes were most prominent in the temporal region, however abnormalities were also found in the left frontal and right parietal cortex. This already suggested a frequency-specific long-range cortical network, however no measure of functional coupling was applied. In another study using Magnetoencephalography to describe power changes in the temporal cortex we showed an increase of gamma band activity in chronic tinnitus patients [19]. However, these changes were only calculated for time windows around slow-wave peaks and we did not investigate long-range coupling. Theoretically, power changes of Neuronal Cell Assemblies and coupling between them can be completely independent [20,21] and thus we were not able to deduce knowledge about inter-regional coupling from these studies.

With the present study we aimed to investigate inter-areal functional coupling of spontaneous activity in tinnitus patients and to compare them with normal controls. Functional coupling was measured in a broad frequency range from 1 to 90 Hz by means of phase locking analysis. We used a phase locking method described by Lachaux et al. [22], which measures the phase difference between two recorded signals to quantify whether this phase difference is constant over time. A perfect coupling of the two signals results in a constant phase difference and is operationalized with a phase locking value of one. Lower values indicate weaker phase locking and the value of zero reflects no phase coupling at all.

## Results

### Global Phase Locking

**Figure 1:**
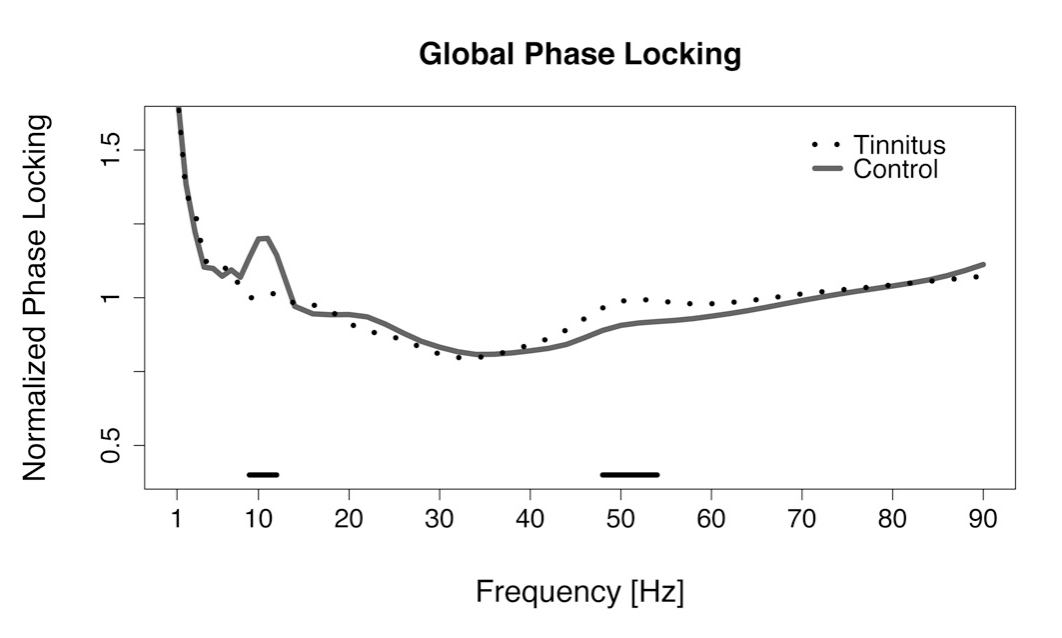
**Phase locking averaged over all connections**. Differences between the tinnitus and the control group were significant in the alpha 9–12 Hz and the gamma 48–54 Hz frequency range. Asterisks mark significant frequency bins after Bonferroni correction.

Our first goal in this study was to identify the frequency bands that displayed group differences in the long-range coupling of cortical activity. The following steps of the analysis were restricted to these frequency bands. Therefore, we averaged the normalized phase locking of all connectivities in order get one general value for each frequency bin and participant. This „Global Phase Locking" value represents the mean phase locking across all long-range connections for a given frequency. Non-parametric tests between groups were calculated for each frequency. To correct for multiple comparison, we adjusted the threshold for a significant group difference to the level of p = 0.00098 according to the Bonferroni-method. We observed a dominant peak of phase coupling in the alpha band (9–12 Hz) for the control group, which was completely absent in the tinnitus group (Figure 1). Furthermore, we found an increase of gamma coupling in tinnitus that spans over a frequency range from 40 to 70 Hz, with the frequency bins in the range from 48 – 54 Hz surviving the Bonferroni correction. Phase locking values per frequency are displayed relative to the mean across all frequencies.

### Analysis of the alpha and gamma network

**Figure 2:**
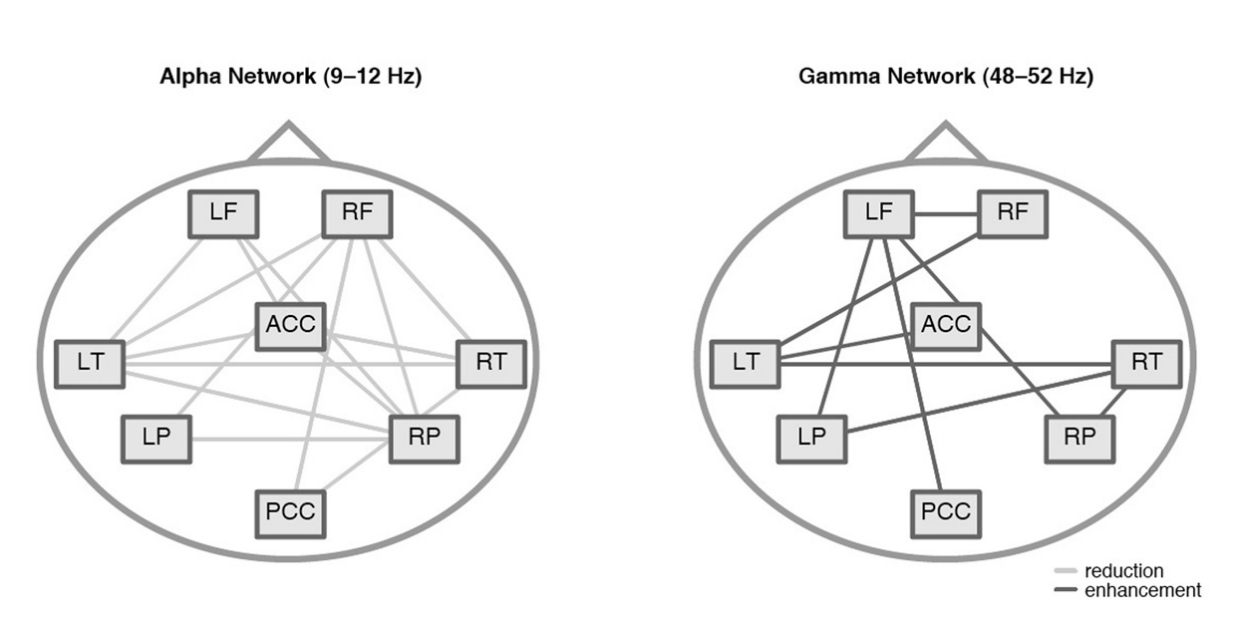
**Schematic display of the alpha and the gamma network. Connections with a significant group difference were plotted as edges in the networks**. The nodes were named by: LF = Left Frontal, RF = Right Frontal, LT = Left Temporal, RT = Right Temporal, LP = Left Parietal, RP = Right Parietal, ACC = Anterior Cingulate Cortex, and PCC = Posterior Cingulate Cortex.

In the following analyses we concentrated on 1) those connections that showed a significant decoupling in the alpha (9–12 Hz) frequency range for the tinnitus group, and 2) connections showing a significant increase in the 48–54 Hz gamma band in tinnitus. The same p-value correction factor as in the previous analysis was applied. Figure 2 shows the architecture of the networks that we will call 'alpha network' and 'gamma network' in the following.

**Figure 3:**
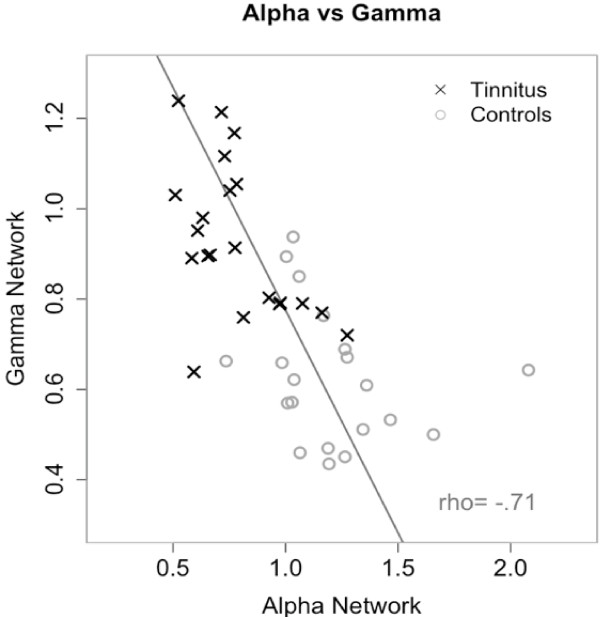
**Negative relationship between the alpha and the gamma network**. As an indicator for the strength of the alpha and the gamma network we calculated the sum of all phase locking values for both frequency bands. Decoupling of long-range alpha is associated with an increase of long-range gamma coupling. Individuals with tinnitus can be separated from controls with an accuracy of 83%.

At the single subject level there was a strong inverse relationship between the alpha- and gamma coupling both in tinnitus patients and in controls. To describe this relationship we calculated an indicator for the alpha and the gamma network activation in each participant. Therefore we added up all phase locking values (not normalized) of the respective connections in the alpha and gamma frequency range respectively. Whenever the alpha coupling was high in a participant, the gamma coupling was low. This resulted in a strong negative correlation of r = -0.71 (p < 0.001) between the networks (Figure 3). Furthermore, the interplay of alpha and gamma discriminated well between participants with tinnitus and those without. In tinnitus patients, alpha coupling was low, while gamma coupling was high. In control participants, alpha coupling dominated while there was a low coupling in the gamma range.

Theoretically it is possible that the negative correlation between alpha and gamma coupling merely reflects an epiphenomenon of the normalization technique that we used here. To test this alternative we repeated the same calculation without normalization and found again a highly significant negative correlation between the alpha and gamma coupling, however with slightly smaller effects (p < .001, rho = -0.57). This still speaks for an inverse relationship between the coupling of the alpha and the gamma frequency network.

From figure 3 one might get the impression that the tinnitus and the control participants are well separated by the alpha and gamma network activation. To test this observation we applied a linear discriminant analysis (LDA) to the data using leave-one-out cross-validation. Starting with the first patient we applied LDA to the data set without the first patient and used the resulting linear discriminant function to classify the omitted patient. This was repeated for every single patient. Using this method we classified 83% (34 out of 41 participants) correctly to their respective group.

### Influence of Tinnitus Duration

Focal stimulation of the temporal cortex by transcranial magnetic stimulation [23-27] or by direct electrical stimulation [28] reduces tinnitus perception. However for both TMS [23-25,27] and epidural stimulation [29,30] of the auditory cortex, tinnitus duration seems to determine treatment efficacy with much better results in patients with less than 3 to 4 years tinnitus duration. This finding of 3–4 years tinnitus duration as a kind of turning point in the efficacy of auditory cortex modulation led us to the assumption that functional architecture may change with increasing duration. Since the median of the tinnitus duration in our sample was 4 years, a median split as a data driven post-hoc stratification allowed us to test whether patients with a tinnitus duration of less than 4 years differ in their functional architecture from those with longer lasting tinnitus. The patient group with tinnitus of shorter duration (4 years or less) consisted of 10 participants (1 female, 9 males; mean tinnitus duration of 2.3 years), the group with longer tinnitus duration (more than 4 years; average: 9.8 years) of 11 patients (4 females, 7 males). The two groups did not differ in age (P > 0.8) nor in tinnitus distress (assessed by the German version of the Tinnitus Questionnaire [31]) for the two groups (P > 0.9).

**Figure 4:**
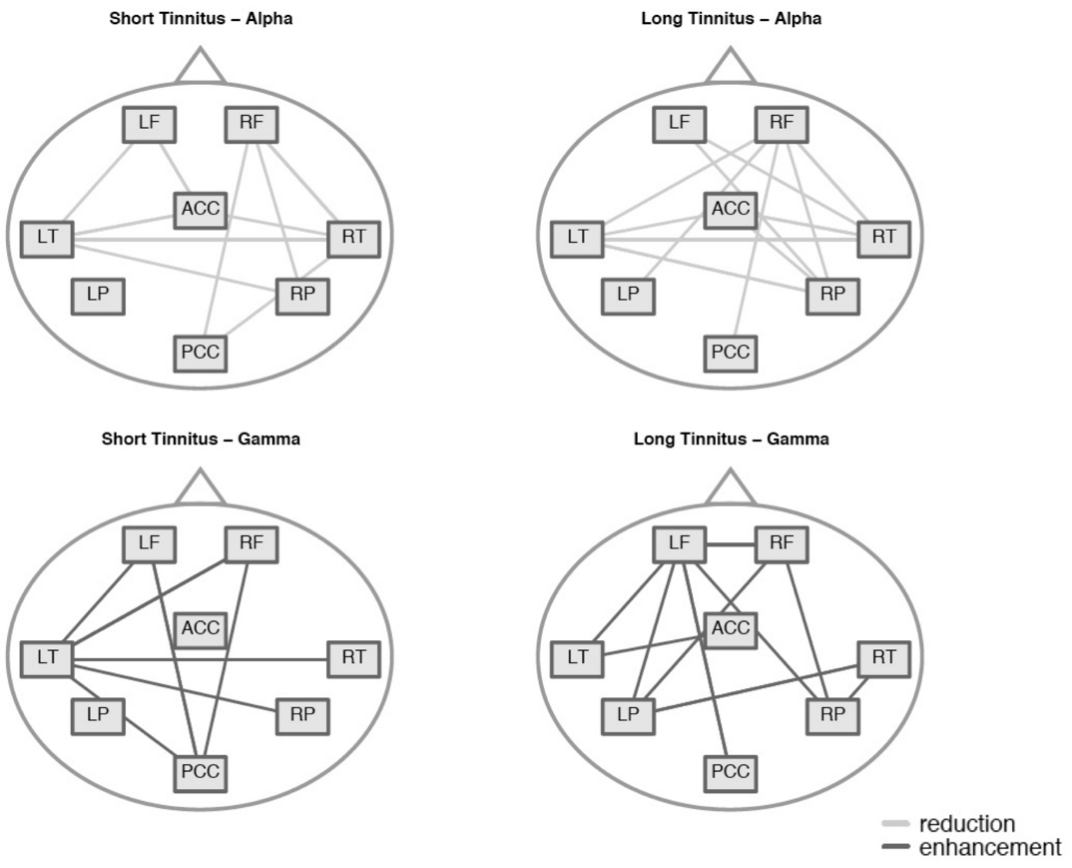
**Schematic display of alpha and gamma networks for both tinnitus subgroups with tinnitus of short and long duration**. LF = Left Frontal, RF = Right Frontal, LT = Left Temporal, RT = Right Temporal, LP = Left Parietal, RP = Right Parietal, ACC = Anterior Cingulate Cortex, and PCC = Posterior Cingulate Cortex.

**Figure 5:**
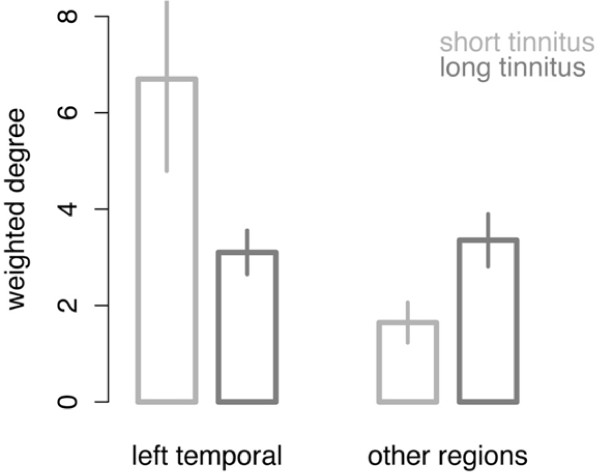
**Centrality of the left temporal cortex in the gamma network**. As a measure for the centrality of the left temporal cortex we calculated the „weighted degree" for both subgroups by adding the number of links that connect to the left temporal source, weighted by the strength of the connections. The centrality of the left temporal cortex was compared with the centrality of other regions. Error bars indicate the standard error of the mean over the respective tinnitus group.

**Figure 6:**
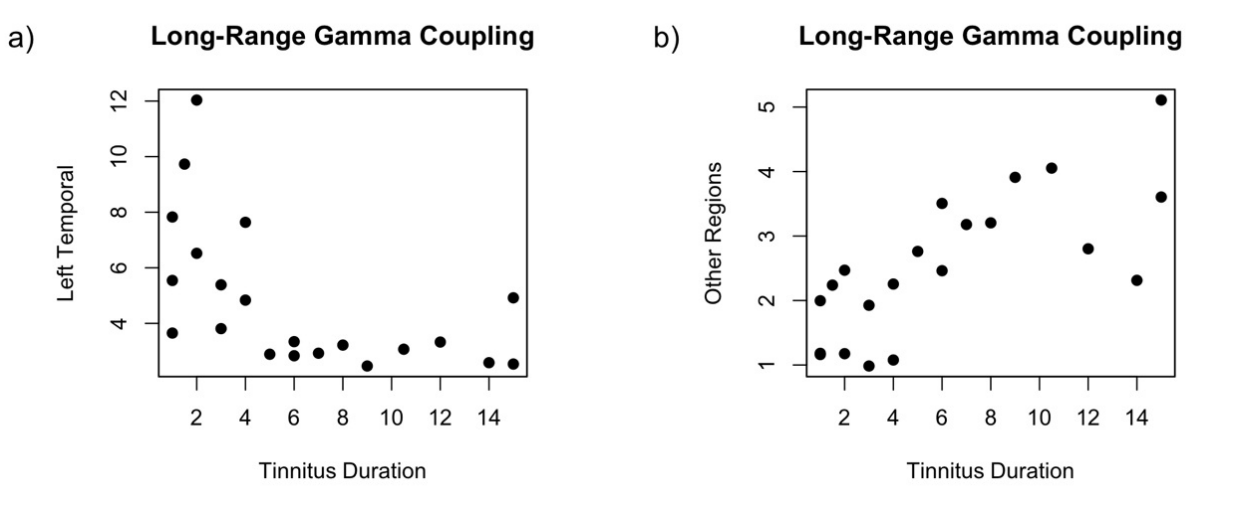
**Relationship between the tinnitus duration and the long-range gamma coupling of the left temporal cortex (r = -.56, p < .01, figure 6a) and the remaining sources (r = .74, p < .001, figure 6b)**.

In the next step, we analyzed the architecture of the alpha and the gamma network in the 9–12 Hz and the 48–54 Hz frequency band, respectively, for both groups. The results in figure 4 show the difference between each tinnitus group (short duration, long duration) and the control group. Regarding the alpha network, there were only marginal differences between the tinnitus subgroups: There was no significant difference in the average alpha coupling between the groups (P > .9) and the architecture of the networks looked about the same. In the average gamma coupling between the tinnitus subgroups there was again no significant difference. However, a closer look to the architecture of the gamma network leads to the impression of a fundamental difference between the subgroups: In the group with short tinnitus duration, the majority of gamma band connections involved the left temporal cortex. Contrary to the group with tinnitus of long duration, the network was more widely distributed across the entire cortex. The left temporal cortex did not play a central role in this subgroup (Figure 4). In order to test this observation statistically we calculated an indicator for the centrality of each node within this gamma network. The 'centrality' of a node is high when a node connects to many other nodes and is low if the nodes connect to only few other nodes. The 'degree of node' is a measure for this centrality and it is calculated by simply adding the number of links that connect to the respective node. With the 'weighted degree' we weighted this number of links by the strength of the connections. We calculated this 'weighted degree' for the left temporal node by adding all phase locking values of all the significant connections going the left temporal source. For the left temporal cortex, the difference in the centrality between the tinnitus subgroups was highly significant (Wilcoxon rank sum test: W = 107, p < .0001, figure 5). The mean centrality of the remaining sources also revealed a strong significant group difference (Wilcoxon rank sum test: W = 108, p < .0001, figure 5) with the long-term tinnitus group having a greater mean centrality. This indicates that in patients with short tinnitus duration, the left temporal cortex plays a central role in the gamma network. However, if the tinnitus is experienced for a longer duration, the centrality (i.e. importance) of the left temporal cortex is reduced and the importance of other regions is increased. With a correlation analysis we investigated the linear relationship between the tinnitus duration and the weighted degree for the temporal cortex (figure 6a) and the other regions (figure 6b). The long-range gamma coupling of the temporal cortex decreased with longer tinnitus duration (r = -.56, p < .01). Remarkably, there was a strong decline after 4 years of tinnitus duration. Contrary, the long-range gamma coupling of the other regions increased almost linearly with longer duration of tinnitus symptoms (r = .74, p < .001).

## Discussion

Here, we first present substantial differences in the resting-state long-range functional coupling in chronic tinnitus sufferers. Specifically, two networks of different architectures and anti-correlated activity primarily account for the group differences. First, tinnitus patients are characterized by a decrease of phase couplings in the alpha frequency. Second, they display enhanced phase coupling in the 48–54 Hz gamma range. In both the tinnitus and the control group, there was a significant negative correlation between the alpha and the gamma network activity, suggesting an interplay of alpha and gamma coupling on an individual level. Furthermore, the duration of tinnitus seems to have an impact on the network architecture. In patients with tinnitus of short duration, gamma network changes are concentrated on the left temporal cortex. In contrast in the group with longer tinnitus duration, this network appears more widespread distributed over the entire cortex with lower impact of temporal areas.

Because the source montage that we used in this study covers only main areas of interest in the cortex, we are not able to interpretation of the precise location of the coupled sources. This is also because of technical constraints that are inherent to the inverse modeling used in MEG. However, the rough coverage of the brain however does not diminish the frequency-specific findings reported here. Also, we analyzed the power spectra of all sources to check whether they match with findings that were reported elsewhere [8]. Power spectra analysis of spontaneous resting-state data (eyes open) in tinnitus usually shows a reduction of alpha power and an enhancement of slow-wave power [8]. In this study, the alpha reduction was most pronounced for the temporal areas and to a smaller amount in the parietal and poster regions. The enhancement of the slow-wave power was localized mainly in the left temporal cortex. Overall, the alpha reduction was stronger than the enhancement of the slow-waves. In the additional material to this paper we report the grand average power spectrum over all sources (additional file 1) and the power spectra of all source locations (additional file 2) of the current analysis. The effects that we found in earlier studies were also observed in this analysis. Additionally, there was a slight increase in gamma power that was also found in another study of our group [32]. Furthermore, the additional file 3 gives a graphical illustration of the alpha power distribution (9–12 Hz) over the sources for the tinnitus and the control group. Occipital alpha power in resting state recordings with eyes open is usually smaller than in recordings with eyes closed. With this source montage, occipital alpha power is largely represented by the PCC-source. There was no significant group difference in the alpha power of this source (P > .2).

In this study, we found evidence for abnormal functionality in long-range cortical networks between tinnitus and control participants in the resting state, which are specific to the alpha and gamma frequency band. A general interaction between alpha and gamma power in the brain has already been postulated earlier [33]. It is assumed that alpha directly or indirectly reflects an intrinsic mechanism that prevents the build-up of gamma coupling within neural cell assemblies during deprivation from input. Functionally, such a mechanism appears to be necessary, as strongly interconnected excitatory oscillators would have a natural tendency to synchronize their activity. A deficiency of this mechanism is putatively an important prerequisite for the emergence of phantom perceptions. Our finding suggests that this relationship between alpha and gamma frequency is not limited to local power changes, but also might apply to inter-areal phase coupling.

We found that the inter-regional coupling of the alpha and gamma frequency bands discriminate well between the tinnitus and the control participants. Participants with a tinnitus perception are characterized by a decrease of long-range alpha coupling and an increase of long-range gamma coupling. Even though the discrimination of 83% is not sensitive enough to use it as an objective diagnostic tool for tinnitus, this is a strong argument that long-range couplings play an important part in the neuronal mechanisms associated with the tinnitus perception.

Here we propose a tinnitus model that integrates this finding with current knowledge on the tinnitus. On a first level the tinnitus is generated within the central auditory system and is most likely a result of reorganization processes triggered by damage to the hearing system. This is supported by numerous studies that show functional reorganization of the auditory system in tinnitus patients [3,8,34-36]. On a second level, abnormal coupling with higher-order brain regions outside the auditory system underlies its conscious perception [9,10]. We assert that both levels are necessary for an ongoing perception of the tinnitus phantom sound.

Even though the alpha and gamma coupling discriminated well between tinnitus and control participants an association between the long-range coupling and the subjective degree of tinnitus distress was lacking. In an earlier study – also with resting-state recordings in the MEG – we found moderate correlations of the subjective tinnitus rating with alpha power decrease in temporal regions [8]. It is likely that we investigated two different neuronal mechanisms: One mechanism that is involved in the general perception of tinnitus and the other mechanism that is associated with tinnitus distress. The former study reported an association between temporal power changes and tinnitus distress. The current study on long-range coupling discriminated well between tinnitus perception and no tinnitus perception. Both mechanisms do not necessarily have to be associated.

With respect to tinnitus duration we found that longer-lasting tinnitus (> 4 years) accompanies marked changes in the pattern of the gamma network compared to shorter-lasting tinnitus. The most obvious difference between long and short-lasting tinnitus is a decrease in importance of the left temporal part of the network, i.e. there are fewer connections formed within this brain region. On the other side, functional connections between non-auditory areas are increased in tinnitus of longer duration. Based on the data that we present here we cannot decide whether this is only a change of functional coupling or whether structural changes also occur. A study using diffusion tensor imaging could help to clarify this question.

Notwithstanding whether the change in the network architecture is structural or functional, the results offer an explanation for a so far unresolved riddle in treating chronic tinnitus with Transcranial Magnetic Stimulation: It has been shown in a series of clinical studies that the efficacy of TMS treatment strongly depends on the duration of tinnitus [24,25,27,37]. In these studies shorter tinnitus duration predicts better treatment outcome for therapeutical application of Transcranial Magnetic Stimulation applied while the treatment efficacy declines with longer duration of tinnitus. A tinnitus duration of 3 to 4 years seems to represent the turning point and patients below this point benefit only little from the treatment. The intriguing detail about this is that TMS is traditionally applied to the left temporal cortex. In the light of our findings these negative treatment effects make sense: as the gamma network shows a major hub in the left temporal cortex of patients with short tinnitus duration, stimulation of this region exhibits a potentially great impact on this network. However, since the gamma network is more widespread in patients with a long history of tinnitus, the impact of the stimulation to the left hemisphere is largely reduced. This idea of an alteration of the tinnitus-related neural network over time was hypothesized earlier [25] and the data that we presented here are the first experimental support for this idea.

## Conclusion

Here we demonstrate for the first time alterations in the long-range network during spontaneous activity in tinnitus patients. The results can be described by an overall decrease of coupling in the alpha frequency band together with an increase of gamma coupling. This pattern of phase coupling discriminates with a high percentage (83%) the tinnitus patients from the healthy controls.

Here we suggest a tinnitus model that incorporates 1) altered activity of the central auditory system that most likely generates the tinnitus sound and 2) the coupling across distant brain regions that is needed for a conscious perception of the tinnitus sound.

## Methods

### Participants

Twenty-one individuals with tinnitus (five female; mean age: 43.6 years; range: 20–65, see table 1) and 20 normal controls (six female; mean age: 35.6 years; range: 23–78) participated in the study. The participants were recruited via newspaper ads and flyers posted at the University of Konstanz. There was no significant difference in age between the tinnitus and the control group (P > 0.1). All tinnitus patients suffered from chronic tinnitus with a mean duration of 6.2 years (range: 1–15). Tinnitus severity, as assessed by a standardized German questionnaire (Tinnitus Fragebogen, [31]), varied in this sample between slight (4 points) and severe (50 points) distress with an average of 23.6 points. The maximum potential score in this Questionnaire is 84 points. All participants were reimbursed after the measurement. Prior to the experiment, all participants gave written informed consent in accordance with the Ethical Committee of the University of Konstanz. The participants were all right-handed according to the Edinburgh Handedness Inventory [38].

**Table 1:** Characteristics of the tinnitus group

Participant	Sex	Age	Tinnitus Distress	Etiology	Tinnitus duration [years]	Tinnitus side
1	M	29	NA	Unknown	1	R
2	M	53	50	Stress	1	B
3	M	63	47	Stress	1	L
4	M	52	20	Sudden hearing loss	1	B
5	F	32	5	Unknown	2	R
6	M	20	32	Unknown	2	R
7	M	23	3	Noise trauma	3	B
8	M	65	21	Unknown	3	L
9	M	60	13	Occupational noise	4	L
10	M	34	18	Sudden hearing loss	4	L
11	M	33	19	Hereditary	5	B
12	F	22	8	Unknown	6	B
13	F	25	4	Unknown	6	L
14	M	61	35	Unknown	7	B
15	M	52	21	Sudden hearing loss	8	L
16	M	26	21	Lyme disease	9	B
17	M	58	22	Stress	11	B
18	M	57	30	Occupational noise	12	R
19	F	38	20	Sudden hearing loss	14	R
20	M	55	45	Mandibular disorder	15	B
21	F	58	38	Stress	15	L

### Data Acquisition

Five minutes of resting activity were recorded using a 148-channel whole-head MEG system (MAGNES 2500 WH; 4-D Neuroimaging, San Diego, USA) installed in a magnetically shielded and sound-proof room (Vakuumschmelze Hanau, Germany). The participants were instructed to lie quietly in a comfortable supine position. Data was recorded with a sampling rate of 678.17 Hz and a hard-wired high-pass filter of 0.1 Hz. The participants were instructed to keep their eyes open and to stay awake without engaging in any specific thought process. To avoid eye and head movements, participants were asked to focus on a spot on the ceiling of the measurement room. Electrooculagramm (EOG) and Electrocardiogramm (ECG) were recorded in parallel to the MEG acquisition.

### Data Analysis

Preprocessing of the continuous data stream was performed in BESA (Brain Electrical Source Analysis, MEGIS Software, Germany) using a semi-automated process for artifact reduction of the eye blinks and the heartbeats [39]. Time segments of muscle artifacts were rejected based on visual inspection and segments of one minute length were selected for further analysis. A source-space projection was applied using the source analysis module in BESA. This projection was not supposed to represent the precise neuroanatomical structures but rather to create a multiple-dipole space of lower dimension – i.e. coarsely defined "brain regions" with a greater signal-to-noise ratio [40]. Thus, a brain montage of eight regional dipoles (left and right temporal plane approximately at the location of Heschl's gyrus, left and right prefrontal area, left and right parietal lobe, one near the Anterior Cingulum, and one near the Posterior Cingulum) with fixed locations was used for each participant and adjusted to the individual head size. The dipole locations were defined prior to the analysis in order to cover roughly the activity of major cortical regions (see supporting information for exact locations).

The data were down-sampled to 450 Hz and one artifact-free minute was selected for subsequent analyses. A Morlet wavelet (m-factor = 7) was used for estimation the instantaneous phases in the frequency range of 1 to 90 Hz (1–12 Hz in steps of one Hz, 14–90 Hz in steps of two Hz) using Matlab 7.4 (The MathWorks, Natick, MA). The phase information over one minute of data was used to calculate the phase locking value (PLV) that has been suggested to be an operationalization for functional coupling [22]. Therefore, the phase difference between two signals is calculated and tested for stability through all time points. The phase locking value (from zero to one) increases the more the distribution within a unit circle deviates from uniformity, with a PLV of one indicating perfect phase coupling between the two signals. To make the data comparable across participants in terms of differential coupling values across the frequency bands, we normalized the phase locking values with respect to frequency. That was done by calculation a mean PLV over all frequencies and dividing all phase locking values by this mean. Thus, the mean „normalized phase locking" over all frequencies equals one for each participant.

Statistical analyses were all conducted in R . Group differences were assessed using the non-parametric Wilcoxon's rank sum test with a Bonferroni correction for multiple comparisons of the fifty-one frequency bins. Correlations between the alpha and gamma PLVs were determined using a Spearman's rank correlation.

## Authors' contributions

WS carried out the study, performed the statistical analysis and drafted the manuscript. TH participated in data acquisition, supplied analysis tools and helped in drafting the manuscript. BL helped in drafting the paper and the discussion of the results. NW participated in the data collection, data analysis and writing the manuscript.

## Supplementary Material

Additional file 1 **Supplemental figure 1**. Grand average of the normalized power spectrum over all sources. Click here for file

Additional file 2 **Supplemental figure 2.** Normalized power spectra for all source locations. Click here for file

Additional file 3 **Supplemental figure 3.** Graphical illustration of the normalized alpha power (9–12 Hz) over the 8 sources. Then diameter of the circle denotes the strength of the alpha power. The average over the tinnitus group is shown on the left side, the control group on the right side of the figure. Click here for file
