# Supplementary material for: Abnormal resting-state cortical coupling in chronic tinnitus
Source: BMC Neurosci. 2009 Feb 19;10:11. doi: 10.1186/1471-2202-10-11 (PMC2649130; doi:10.1186/1471-2202-10-11)
Supplement: Additional file 1 — Supplemental figure 1. Grand average of the normalized power spectrum over all sources. [file 1471-2202-10-11-S1.pdf]

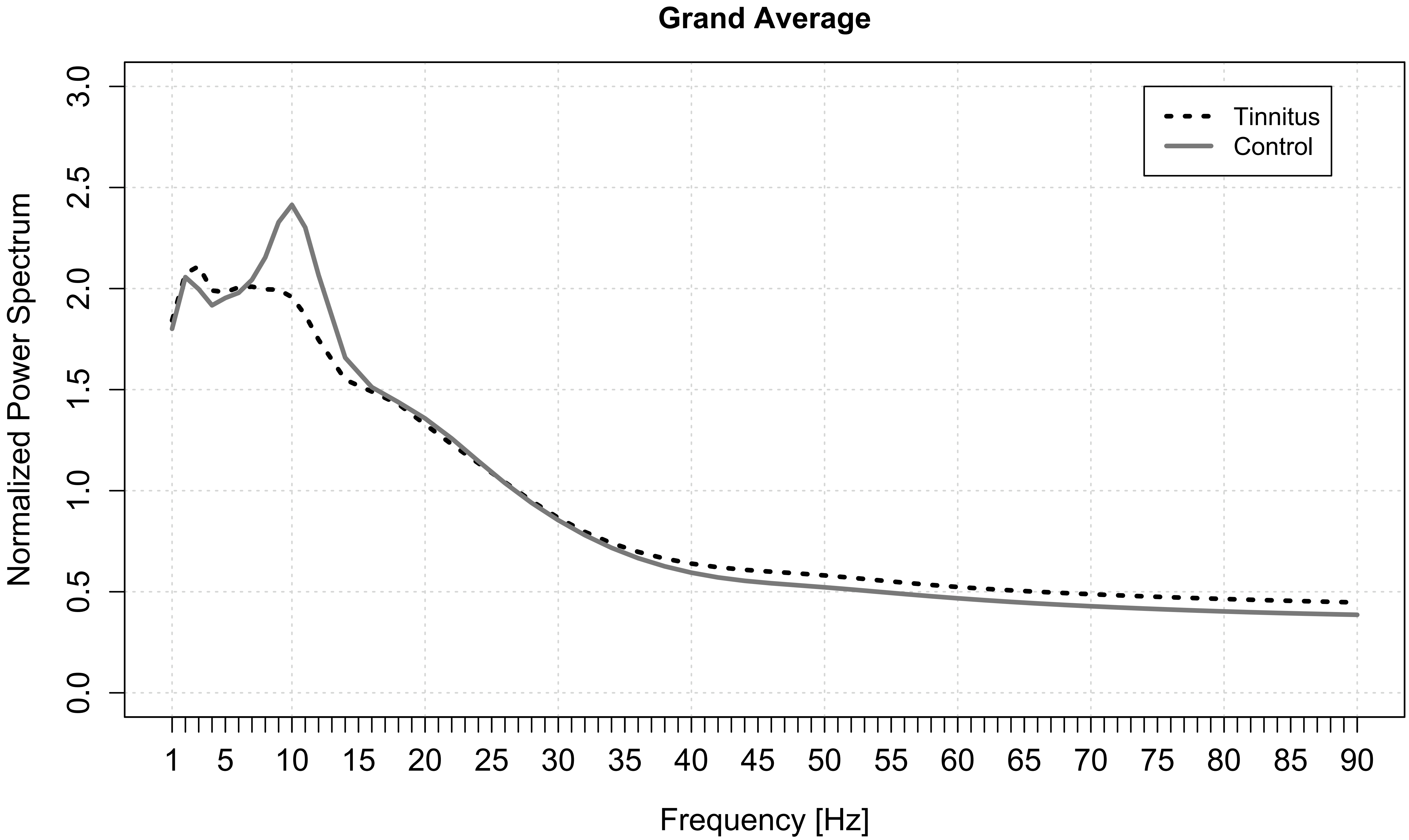

Additional File 1.
Grand average of the normalized power spectrum over all sources.
