# Supplementary figures and images for: Abnormal resting-state cortical coupling in chronic tinnitus
Source: BMC Neurosci. 2009 Feb 19;10:11. doi: 10.1186/1471-2202-10-11 (PMC2649130; doi:10.1186/1471-2202-10-11)

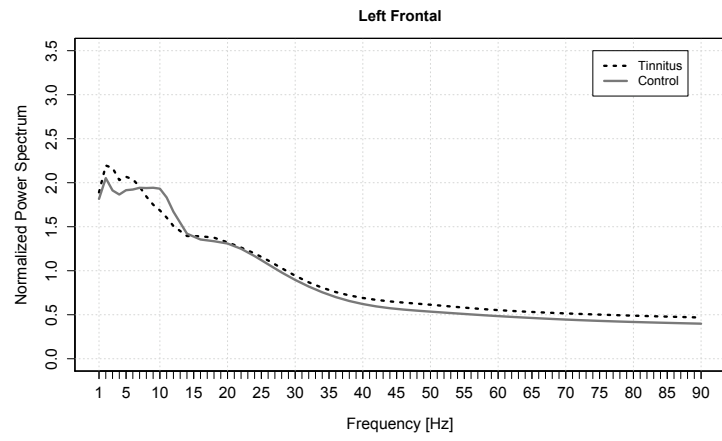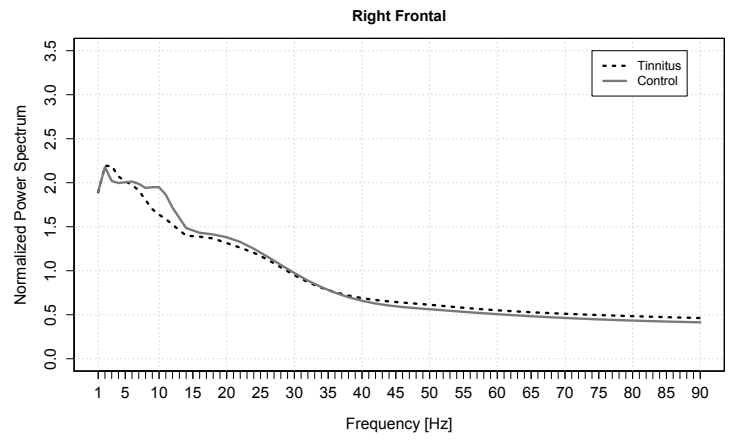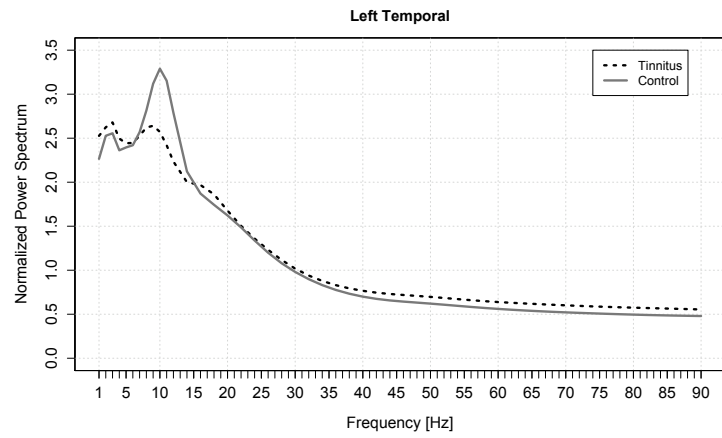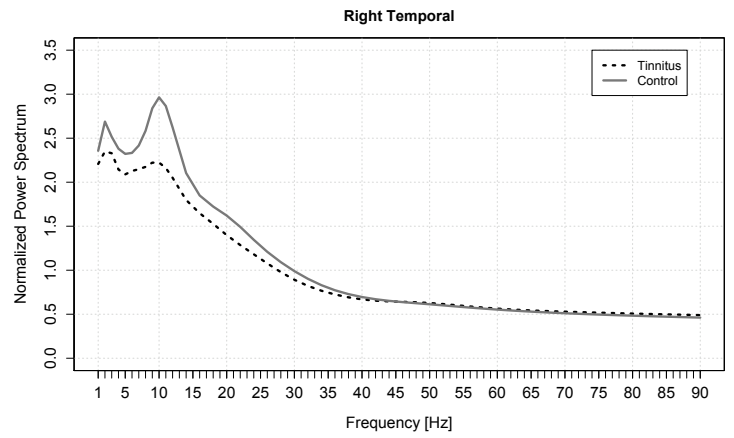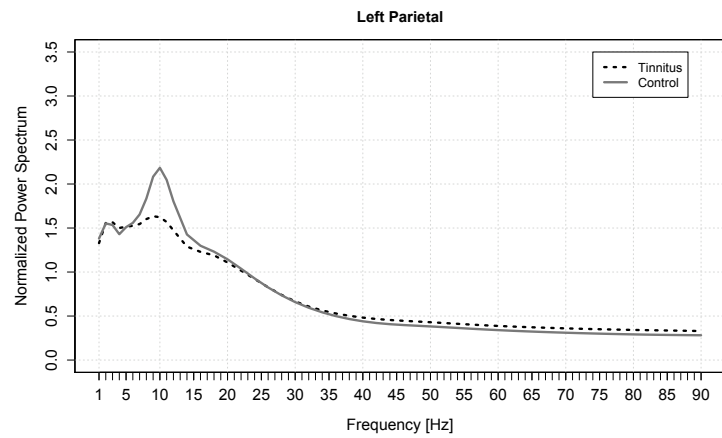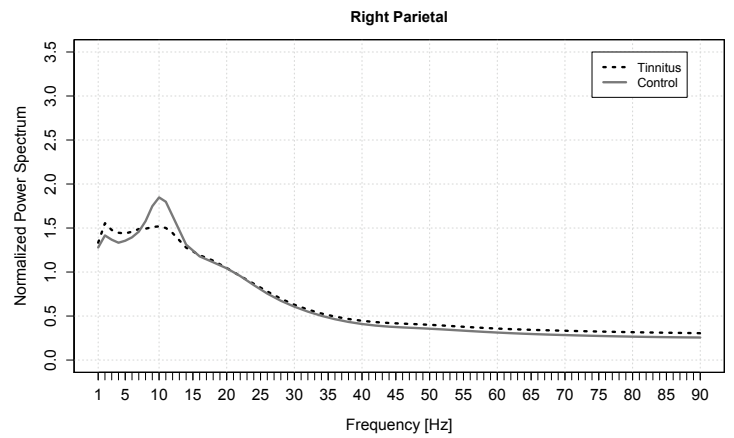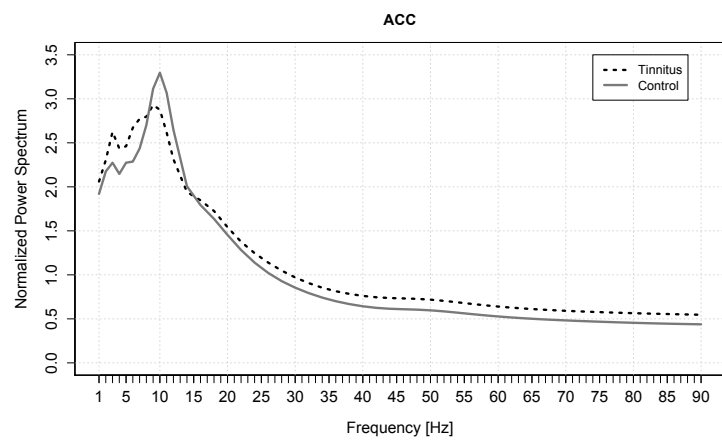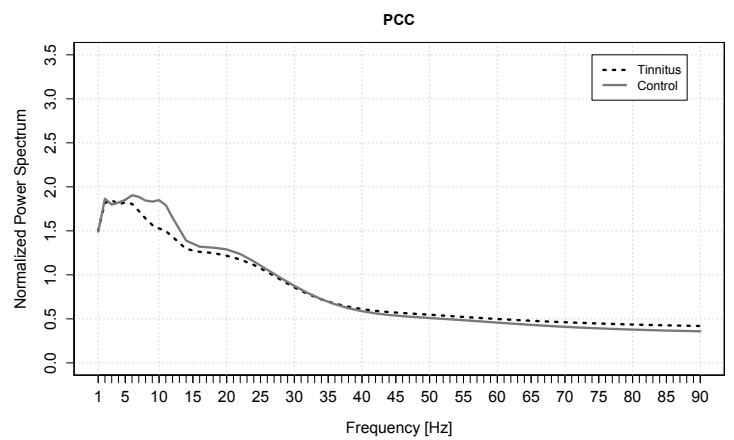

**Additional File 2.**  
Normalized power spectra for all source locations.

Supplement: Additional file 2 — Supplemental figure 2. Normalized power spectra for all source locations. [file 1471-2202-10-11-S2.pdf]
