# Supplementary material for: Abnormal resting-state cortical coupling in chronic tinnitus
Source: BMC Neurosci. 2009 Feb 19;10:11. doi: 10.1186/1471-2202-10-11 (PMC2649130; doi:10.1186/1471-2202-10-11)
Supplement: Additional file 3 — Supplemental figure 3. Graphical illustration of the normalized alpha power (9–12 Hz) over the 8 sources. Then diameter of the circle denotes the strength of the alpha power. The average over the tinnitus group is shown on the left side, the control group on the right side of the figure. [file 1471-2202-10-11-S3.pdf]

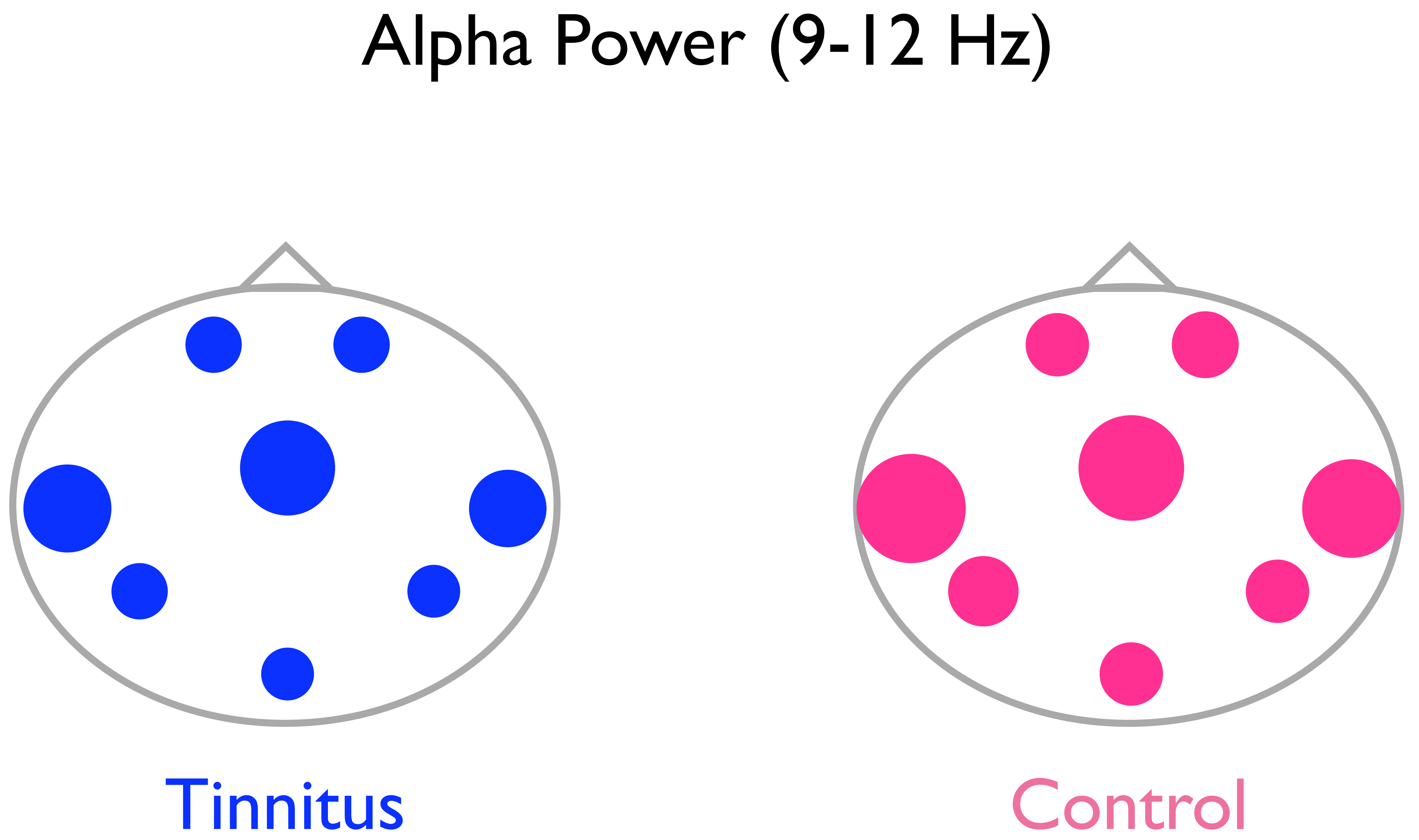

Additional File 3.
Graphical illustration of the normalized alpha power (9-12 Hz) over the 8 sources. The diameter of the circle denotes the strength of the alpha power. The average over the tinnitus group is shown on the left side, the control group on the right side of the figure.
